# Local large language model‐guided optimization for prostate radiotherapy planning

**DOI:** 10.1002/mp.70654

**Published:** 2026-09-10

**Authors:** Humza Nusrat, Bing Luo, Hassan Bagher‐Ebadian, Ryan Hall, Joshua Kim, Anthony Doemer, Khaled Adil, Mohamed Elsheikh, Aharon Feldman, Benjamin Movsas, Kundan Thind

**Affiliations:** ^1^ Department of Radiation Oncology Henry Ford Health Detroit Michigan USA; ^2^ College of Human Medicine Michigan State University East Lansing Michigan USA

**Keywords:** artificial intelligence, autonomous planning, large language models

## Abstract

**Background:**

Radiotherapy planning is time‐intensive, iterative, and operator‐dependent. Locally deployed large language‐model agents may support planning automation while avoiding transmission of patient data to external model providers.

**Purpose:**

The purpose of this work was to develop and evaluate SAGE, a locally deployed LLM agent for autonomous VMAT optimization‐priority tuning, and to investigate the impact of model size (8B vs. 70B) and intra‐case memory implemented through retrieval augmentation.

**Methods:**

The SAGE agent (Llama 3.1 8B/70B) was interfaced with a clinical TPS to tune optimization priority values for 17 retrospective prostate cancer patients (60 Gy/20fx). All SAGE priority values were initialized to 1, while the study‐defined optimization objective set and beam geometry were held fixed. We compared four agent variants (8B, 70B, 8B‐RAG, 70B‐RAG) to the clinical plan using paired Wilcoxon tests with FDR control.

**Results:**

No statistically significant difference in target (PTV V58.5 Gy) coverage was observed between SAGE variants and clinical plans. The 70B‐RAG agent significantly improved penile bulb sparing (V22Gy) over clinical plans (*p* = 0.014). Enabling intra‐case memory provided a statistically significant improvement in penile bulb sparing, while no significant effect was observed from model size alone. While clinical plans were statistically better for some OARs, all SAGE 70B‐RAG plans remained clinically acceptable. The most complex version of SAGE (70B‐RAG) completed ten autonomous iterations in a median compute time of 45 min (IQR: 34–51). For reference, the clinical planning workflow turnaround (ARIA‐recorded task duration) median time was 2700 min (IQR: 2040–3060).

**Conclusions:**

This proof‐of‐concept study demonstrates that a local LLM agent can autonomously tune optimization priority values to generate clinically acceptable prostate VMAT plans within a fixed, study‐defined objective space. Intra‐case memory produced a statistically significant, endpoint‐specific improvement (penile bulb V22Gy). These findings suggest that smaller, efficient models may be viable for constrained treatment‐planning optimization tasks.

## 1 INTRODUCTION

Radiotherapy treatment planning is a complex and crucial task in modern cancer care, where certified dosimetrists are responsible for the treatment planning process. Due to the manual nature of planning, variations can occur due to expertise, availability, and case complexity.^1^, ^2^ We present a locally deployed language model‐based planning agent: SAGE‐RT (Secure Agent for Generative dose Expertise in Radiotherapy Treatment; henceforth referred to as SAGE). Similar to real dosimetrists, SAGE is capable of evaluating structured dosimetric feedback, reasoning over clinical goals, and iteratively adjusting optimization priority values.

There have been significant previous works on the use of artificial intelligence (AI) in radiotherapy treatment planning.^3^, ^4^ These studies relied mainly on neural networks that were trained and then evaluated on the local institution's data. Despite many successful attempts, these traditional AI based planning techniques suffered from several common pitfalls. Firstly, these models were effective planning solutions for the tumor site that they were trained on, limiting their generalizability across the cancer disease spectrum.^5^ Secondly, to non‐technical clinicians, these solutions were “black boxes”. They required technical expertise from the researchers themselves to train, modify, and use these solutions.^6^ Lastly, many of these models were limited in their training and evaluation due to healthcare data regulations.^7^

Since 2023, large language models (LLMs) have been evaluated for use in healthcare.^8^, ^9^, ^10^ In radiation oncology, the use of LLMs has included knowledge evaluation,^11^, ^12^, ^13^, ^14^ segmentation,^15^ and clinical text extraction.^16^ LLM‐based agents have also recently been explored for automated treatment planning and plan optimization.^17^, ^18^, ^19^, ^20^, ^21^ These studies demonstrate the promise of LLM‐based planning workflows, including multimodal plan evaluation, historical plan retrieval, memory mechanisms, optimization trajectory analysis, and prior‐iteration feedback. Many of these systems rely on proprietary or cloud‐hosted frontier models, which can introduce institutional data‐governance concerns when patient information is transmitted outside the local clinical environment.

Beyond data governance, one significant barrier to clinical integration of LLM agents is inference latency. The computational time required for response generation typically scales with model size and complexity, hindering the utility of large‐scale models in time‐sensitive clinical workflows.^22^, ^23^, ^24^ Consequently, smaller, more computationally efficient models are often proposed as viable alternatives for point‐of‐care applications. However, smaller models may have more limited contextual awareness and planning memory than larger models. Intra‐case memory, implemented here through retrieval augmentation, is one strategy to mitigate this trade‐off by allowing the agent to retrieve prior priority choices and resulting dosimetric outcomes from the same active optimization session.^25^, ^26^

The literature highlights the need for interactive planning systems that are clinically transparent, locally deployable, and able to reason over iterative dosimetric feedback. This study introduces SAGE‐RT to evaluate whether a locally deployed, open‐weight LLM agent can autonomously tune optimization priority values to generate clinically acceptable prostate VMAT plans under fixed beam geometry and fixed study‐defined objective definitions. We specifically investigate the impact of model scale and intra‐case memory using a controlled 2 × 2 ablation, testing whether memory augmentation improves plan quality and whether a smaller model can perform competitively in this constrained optimization setting.

## 2 METHODS

### 2.1 Patient cohort and treatment planning

Treatment data for prostate cancer patients being treated within the “*Institution Name—removed as per instructions”* between 2022 and 2024 as per the Prostate Fractionated Irradiation Trial (PROFIT^27^ ^)^ protocol (60 Gy/20fx) was retrospectively obtained. This phase III trial protocol randomized men with intermediate risk prostate cancer to either a hypofractionated course (60 Gy in 20 fx) or a conventional course (78 Gy in 39 fx) of RT. This trial was conducted with rigorous dosimetry requirements. CT images, segmented structures, treatment plans, and dosimetric information for 17 patients were utilized as our patient cohort. All components of this work were done after institutional review board approval. All retrospective treatment plans and SAGE‐generated plans were housed within the Varian Eclipse Treatment Planning System (TPS; V16.1). AAA (V15.6.06) was used for dose calculation (dose grid resolution of 2 mm^3^). Beam geometry was kept fixed to the clinical plan's beams for each patient. Additionally, DVH estimation algorithm (15.6.05) and optimization (15.6.05) were used for all cases. In Eclipse, the treatment planning system (TPS) application (V16.1) and the individually commissioned dose‐calculation, DVH‐estimation, and optimization algorithms carry independent version identifiers; the algorithm versions reported here (AAA 15.6.06; DVH estimation and optimization 15.6.05) are the versions clinically commissioned and in routine use at our institution, and were applied identically to all clinical and SAGE‐generated plans.

### 2.2 SAGE architecture

**FIGURE 1:**
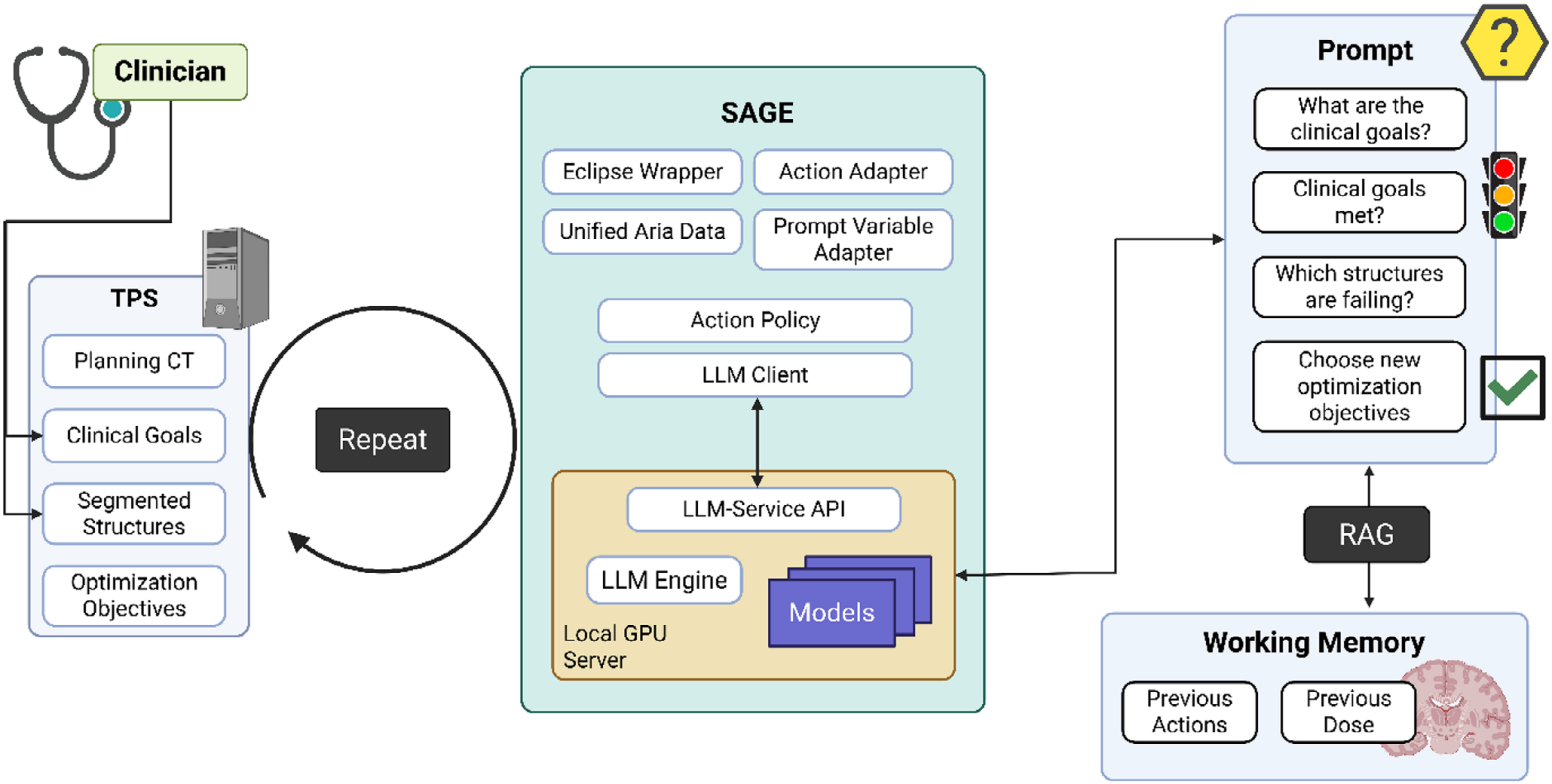
System architecture illustrating the integration and workflow of SAGE. The TPS interacts with SAGE via ESAPI. Within SAGE, there are several coupled components which facilitate the LLM to evaluate and choose optimization priority values. SAGE can access its own previous priority choices and resulting dose metrics through intra‐case memory.

SAGE consists of three interconnected components working in concert to optimize treatment plans (Figure 1). The working memory module maintains the current state of the treatment plan and, when configured, retrieves prior optimization attempts from the same active patient session to inform decision‐making. The LLM serves as the decision‐making engine, analyzing the plan state and proposing adjustments to optimization priority values. Finally, the TPS interface tools enable the LLM to interact directly with the Eclipse TPS by modifying the priority values of optimization objectives. To support local data governance, we deployed LLMs within the hospital's secure computational infrastructure. The LLM engine manages the loading of models and performs inference tasks using in‐house GPUs. A virtualized LLM inference engine encapsulates LLM functionality behind an application programming interface (API). SAGE consists of several coupled components designed for autonomous operation, including the client (prompt formatting), action policy (which guides decision‐making above the client), and action adapter (API connection to TPS). Plan edits were issued via Varian's Eclipse Scripting API (ESAPI) and were restricted to modifying priority values within the study‐defined optimization objective set. Before any SAGE optimization runs, the clinical planning study team translated PROFIT protocol endpoints and institutional prostate VMAT planning conventions into a fixed set of optimizer objective definitions. These objective definitions were not generated by SAGE and were not exported, copied, or tuned from patient‐specific clinical optimizer objective lists. The same objective schema was applied consistently across patients, subject to patient‐specific structure availability. The study‐defined objective types, dose‐volume constraint levels, structure assignments, machine parameters, and beam geometry (arc angles, collimator rotation, isocenter position) remained fixed across SAGE iterations.

Clinical plans were used as dosimetric reference plans only. Their optimizer objective lists and final priority values were not copied, imported, or used to initialize SAGE. Because the retrospective clinical plans were generated with planner‐specific optimization objective sets, clinical and SAGE priority values are not directly comparable as standalone numerical quantities. For every SAGE‐generated plan, the study‐defined optimization objectives were initialized with priority values of 1 before the agent began optimization, creating a neutral priority vector. SAGE then autonomously modified only these priority values over ten iterations based on structured DVH feedback and protocol pass/fail status. Thus, this study evaluates autonomous priority‐weight optimization within a fixed, study‐defined objective space, rather than full de novo plan generation from beam placement and objective construction.

The LLM receives structured text input at each iteration: TPS‐derived DVH values for each structure, protocol pass/fail status computed programmatically from those DVH metrics, current priority values for each optimization objective, and, when intra‐case memory is enabled, up to the three most recent prior optimization attempts from the same patient session, including priority changes, resulting DVH metrics, and remaining failed or near‐failed goals. The prompt encoded a fixed clinical prioritization policy: PTV V58.5 Gy ≥95% was treated as mandatory, and OAR sparing was prioritized after target coverage was stable. For this study, SAGE‐generated priority values were constrained to integer values from 1 to 200. The agent was instructed to return structured JSON containing clinical‐goal status, goals met, priority updates, constraints to monitor, rationale, and confidence. Clinical‐goal status in the agent output was used for structured reasoning and logging; TPS‐derived DVH metrics and programmatically computed pass/fail labels remained the source of truth for dosimetric analysis. LLM outputs were parsed by the action adapter, and only valid priority updates for existing objective IDs were applied. Objectives omitted from the priority‐update list retained their current priority values. Invalid JSON, out‐of‐range priority values, and updates for nonexistent objective IDs were rejected by the action adapter and were not issued to ESAPI. All SAGE sessions completed the full ten optimization iterations autonomously, without manual intervention. The per‐event frequency of adapter rejections (invalid JSON, out‐of‐range priority values, or updates to nonexistent objective IDs) was not systematically logged in this study. The plan generated at completion of the ten‐iteration SAGE session was exported for dosimetric analysis. A representative SAGE optimization trajectory, including priority updates and DVH evolution across ten iterations for one case, is provided in Table S1. No image‐based or multimodal inputs are used. Incorporating multimodal inputs represents a future direction that could provide the agent with spatial dose awareness.

To improve methodological transparency, the representative SAGE prompt template is provided as supplementary material. Key experimental settings, including model configuration, priority‐value initialization, fixed objective definitions, fixed beam geometry, iteration limit, and per‐patient memory reset, are described in the Methods.

### 2.3 LLM

The Llama 3.1 LLM was utilized.^28^, ^29^ Two variants were tested: the 8 billion (8B) parameter model and the 70 billion (70B) parameter model. During all experiments, LLM hyperparameters were kept constant (top_k = 2; T = 0.4). These parameters were optimized in a previous study by our group.^17^ When intra‐case memory was enabled, SAGE could retrieve its own past priority number choices and their resulting doses for the same patient only. This memory store was reset for each patient and did not persist across sessions. These models were housed locally on eight NVIDIA A100 graphical processing units (GPUs) within an on‐site high performance computing cluster.

### 2.4 Statistics and experimental design

For each of the 17 patients, five plans were tested: the clinically used, physician approved plan, as well as the four SAGE variants (8B, 8B‐RAG, 70B, 70B‐RAG). Prespecified dosimetric endpoints (PTV V58.5 Gy, rectum V60Gy, V20Gy; bladder V60Gy, V40Gy, and penile bulb V22Gy) were used for statistical analysis. Paired, non‐parametric Wilcoxon signed‐rank tests were used for all plan‐to‐plan comparisons (*p* < 0.05). The normality of paired differences was statistically assessed using the Shapiro‐Wilk test (with most comparisons yielding *p* < 0.05). This was further supported through visual inspection of Q‐Q plots, confirming the non‐normal distribution and justifying the selection of a non‐parametric approach in this study.

For this study, a plan was operationally defined as clinically acceptable if it met all prespecified PROFIT protocol endpoints, or, for an endpoint not met, if the same deviation was also present in the corresponding physician‐approved clinical plan for that patient (indicating an anatomy‐driven limitation accepted in routine clinical practice rather than a planning failure).

Multiplicity was assessed using Benjamini‐Hochberg (BH) false discovery rate (FDR) (*q* < 0.05). Adjustments were applied within predefined families of related hypotheses, not across all tests. These included: 1) clinical (clinical vs. 8B (least sophisticated variant), clinical vs. 70B‐RAG (most sophisticated variant)), 2) size (8B vs. 70B, 8B‐RAG vs. 70B‐RAG), and 3) memory (8B vs. 8B‐RAG, 70B vs. 70B‐RAG). Data were visualized using violin plots per endpoint with jittered patient points, IQR boxes, and median values. Colored significance brackets were only used in comparisons in which passed within‐family BH criteria. Statistical comparisons and visualization were done using R (tidyverse, ggsignif, and ggbeeswarm).

To understand the influence of intra‐case memory and model size on SAGE performance, within patient main effect Wilcoxon signed‐rank tests were used. For each patient, deltas were computed for model size effect (70B vs. 8B; 70B‐RAG vs. 8B‐RAG). The average of per patient differences was computed and tested against zero with a one sample Wilcoxon test. The same process was repeated to assess the memory effect (70B vs. 70B‐RAG; 8B vs. 8B‐RAG).

Planning times (min) across the four SAGE variants and the clinical plans were computed and depicted using box plots. Planning time includes the time it took SAGE to complete a full cycle (assess, decide on optimization parameters, optimization, dose calculation) for ten iterations. Clinical planning times include task duration in ARIA during working hours (weekdays, 8 h/day). Boxes show IQR, horizontal lines mark medians, and whiskers extend to 1.5× IQR. Points beyond the whiskers are plotted as outliers. Since planning time was not a primary endpoint, analyses are descriptive, and no hypothesis tests were planned.

## 3 RESULTS

### 3.1 SAGE performance

In Table 1, the dosimetry scorecard from PROFIT,^27^ as well as the performance of all plans (the four SAGE variants and the clinical plans) are listed. Values in the cells represent the dosimetric mean and standard deviations from 17 patients. The best performing SAGE plan has been bolded in each row; best was assessed based on mean values only. All four SAGE variants met PROFIT thresholds, except for rectum V60Gy and bladder V60Gy. Of note, these thresholds were not met in the clinical plans either. The 70B‐RAG variant achieved clinically acceptable PTV coverage in all 17 patients, compared to 16/17 for the 8B, 8B‐RAG, and 70B variants, with the most severe failure being concentrated in the smaller model.

**TABLE 1:** Dosimetric data from the clinically treated plans compared to four variants of SAGE.

	DVH	PROFIT	Unit	Clinical	8B	70B	8B‐RAG	70B‐RAG
**PTV**	**V58.5** **Gy**	>95%	%	98.2(±2.0)	95.0(±16.1)	97.6(±3.1)	96.4(±6.8)	**97.9(** ±3.3)
**Bladder**	**V40Gy**	<50%	%	22.8(±19.7)	27.6(±23.5)	**23.4(** ±20.0)	24.9(±19.8)	24.7(±20.4)
	**V48Gy**	<25%	%	15.5(±11.5)	20.1(±18.4)	**16.0(** ±12.7)	16.9(±12.3)	16.6(±12.8)
	**V56.8** **Gy**	<5%	%	9.6(±6.6)	12.6(±11.6)	10.1(±7.3)	**9.9(** ±6.3)	10.3(±7.3)
	**V60Gy**	<1%	%	6.7(±4.6)	9.2(±10.0)	7.8(±5.6)	**6.5(** ±4.4)	8.1(±5.4)
**Femur Head Left**	**Dmax**	<37 Gy	Gy	29.1(±5.1)	31.2(±5.1)	**29.5(** ±4.3)	29.9(±4.9)	30.3(±5.4)
	**V35Gy**	<5%	%	0.0(±0.0)	0.1(±0.5)	**0.0(** ±0.1)	**0.0(** ±0.1)	**0.0(** ±0.1)
**Femur Head Right**	**Dmax**	<37 Gy	Gy	30.1(±4.7)	32.0(±7.1)	30.3(±4.7)	**29.8(** ±5.2)	30.3(±5.5)
	**V35Gy**	<5%	%	0.1(±0.3)	0.8(±2.3)	**0.0(** ±0.2)	0.1(±0.2)	0.1(±0.3)
**Penile Bulb**	**V22Gy**	<50%	%	44.8(±19.6)	42.7(±19.6)	41.0(±19.7)	**39.5(** ±16.9)	39.9(±18.3)
**Rectum**	**V20Gy**	<85%	%	63.2(±15.3)	74.3(±15.9)	**63.5(** ±16.7)	72.1(±15.5)	72.0(±15.0)
	**V30Gy**	<57%	%	42.6(±13.7)	54.3(±19.9)	**41.6(** ±16.7)	49.7(±16.9)	47.7(±17.2)
	**V40Gy**	<38%	%	26.2(±10.7)	32.9(±18.9)	**24.2(** ±12.4)	29.1(±12.9)	27.3(±12.9)
	**V50Gy**	<22%	%	15.3(±6.8)	19.2(±15.0)	**14.4(** ±8.7)	16.6(±8.4)	15.5(±8.8)
	**V60Gy**	<1%	%	5.1(±3.2)	7.5(±8.7)	5.6(±4.8)	**4.0(** ±3.8)	5.6(±4.0)

*Note*: Mean values for the clinical plan and SAGE are listed along with the associated standard deviation in brackets. Numbers highlighted in bold indicate the best (based on mean values only; standard deviation not considered) performing SAGE variant for that particular dosimetric variant. Formal statistical testing was limited to prespecified endpoints (as per Section 2.4).

**FIGURE 2:**
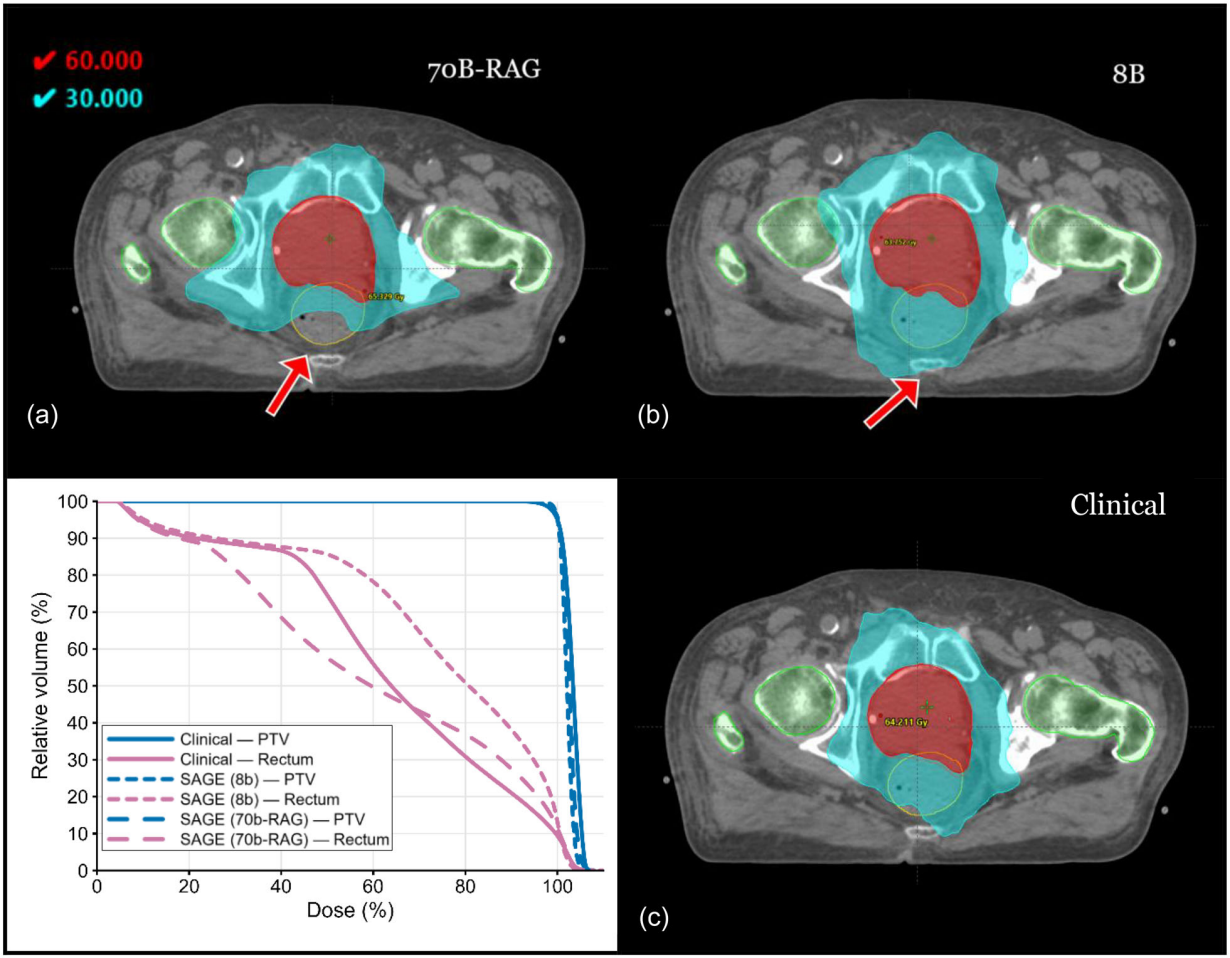
Isodose distributions displayed on an axial slice of a complex prostate case in our cohort. In this case, the PTV overlapped with the rectum volume leading to poorer performance using the 8B parameter model compared to the larger model with intra‐case memory enabled. The 30 Gy (blue) and 60 Gy (red) isodose lines are shown for all three scenarios. Below each axial screenshot, the corresponding DVH is shown (relative to the clinical plan). The 70B‐RAG slice is shown in (a), the 8B SAGE in (b), and the clinical slice in (c).

Figure 2 depicts the performance of SAGE on a complex patient's anatomy. In this case, the PTV and rectum overlapped, leading to difficulty during optimization. The least sophisticated SAGE variant (Figure 2b; 8B only) struggled to push the 30 Gy isodose line away from the rectum, while the advanced SAGE variant (Figure 2a; 70B‐RAG) did. The clinical plan used for this patient is also shown (Figure 2c). The three plans are compared in the dose volume histogram in the bottom left corner of Figure 2.

### 3.2 Comparison of clinical plans to SAGE

**FIGURE 3:**
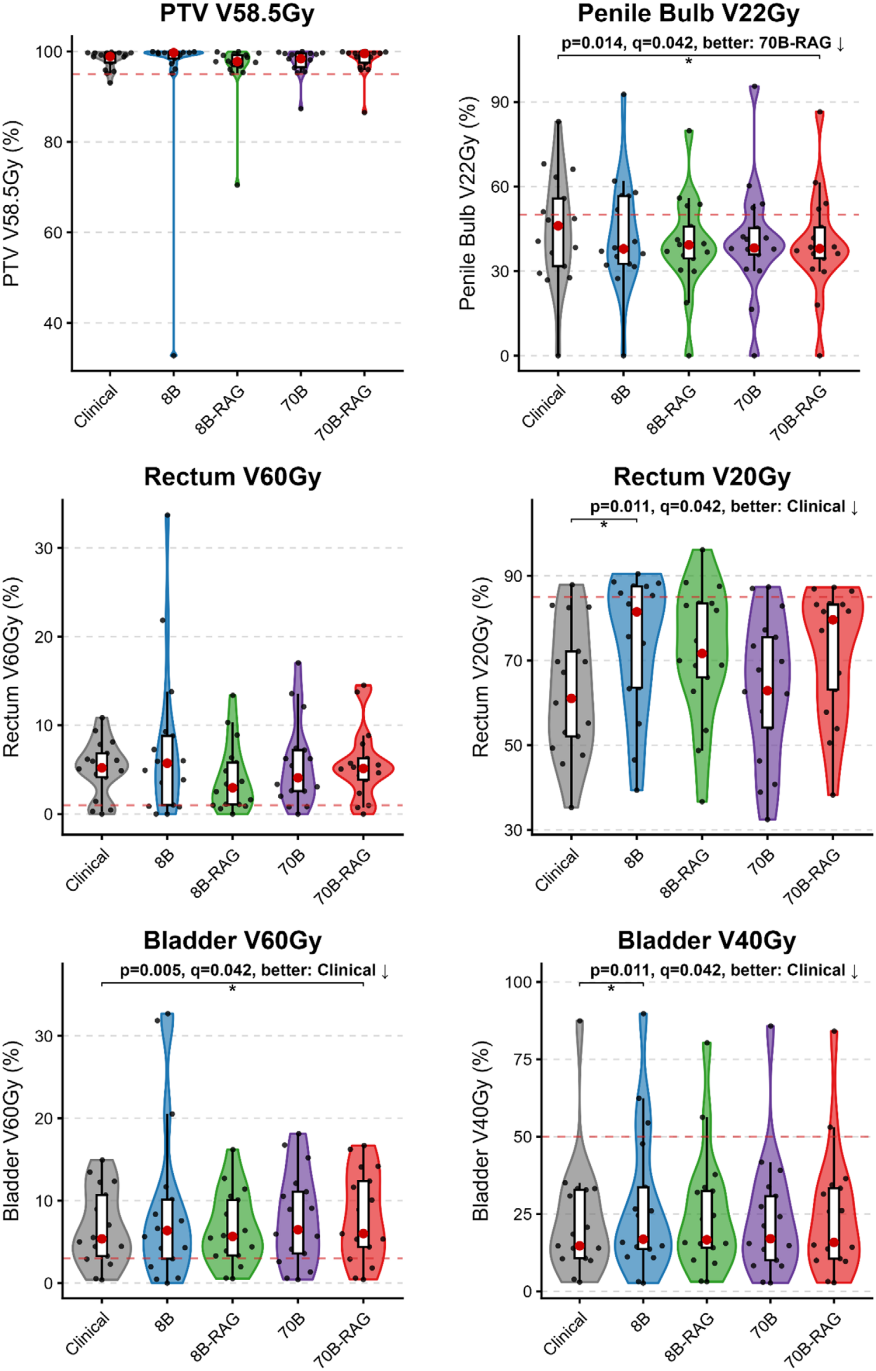
Per patient distributions showing for five plan types (clinical, 8B, 8B‐RAG, 70B, 70B‐RAG). Distributions are shown for all prespecified dosimetric endpoints. Violin represents kernel density, white box represents the IQR, median is represented by the red dot, black dots represent individual patient data points for each variant. Differences in the “Clinical” family (clinical vs. 8B; clinical vs. 70B‐RAG) were assessed for significance. Brackets denote paired Wilcoxon differences that remain significant after within‐family (clinical only) Benjamini‐Hochberg FDR control; associated p and q values for each test are denoted in the top right corner of each subplot. Dashed red lines denote PROFIT thresholds.

Pairwise comparisons are depicted as violin plots in Figure 3. Clinical plans (grey), 8B (blue), 8B‐RAG (green), 70B (purple), and 70B‐RAG (red) are shown for each pre‐selected DVH endpoint. Statistical comparisons were done using Wilcoxon signed‐rank tests with multiplicity control as described in Section 2.4. In each subplot, the violin represents kernel density, the inner white box represents the IQR, and the median is represented by a red dot. Black dots in and around each respective violin represent individual patient data points for each variant. Dashed red lines are used to depict PROFIT thresholds. No statistically significant differences in PTV coverage were observed. The advanced SAGE variant (70B‐RAG) achieved a statistically significant reduction in dose to the penile bulb (V22Gy) compared to the clinical plans. For dosimetric endpoints of rectum V20Gy, bladder V60Gy, bladder V40Gy, SAGE plans were well within PROFIT thresholds, despite the clinical plans being statistically better than SAGE plans. It is important to note that bladder V60Gy threshold (< 1%) was not met by SAGE or the clinical plans.

### 3.3 Evaluation of intra‐case memory and model size on SAGE performance

**FIGURE 4:**
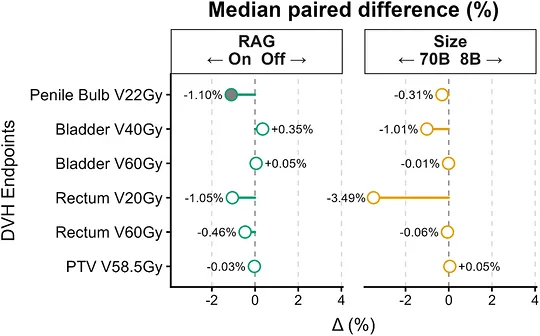
Forest plot of within‐patient paired differences for intra‐case memory and model size on prespecified DVH endpoints. Effect sizes are shown as median paired differences (%). The analysis is based on Wilcoxon signed‐rank test of per patient deltas. The center line indicates no effect. Filled points indicate BH‑significant differences (*q* ≤ 0.05).

The impact of intra‐case memory and model size on SAGE performance was assessed using Wilcoxon signed‐rank tests with multiplicity control. Within‐family comparisons (memory family and Size family, as described in Section 2.4) are shown in Figure 4. This forest plot depicts each prespecified DVH endpoint and the within‐patient change associated with intra‐case memory or model size. The midline represents no change; points to the left of 0% indicate a reduction in dose and vice versa. Filled points indicate statistical significance. Enabling intra‐case memory resulted in a statistically significant reduction in dose to the penile bulb (V22Gy). No other significant differences were observed for the remaining endpoints, although dose sparing was observed for some OARs with higher model size and with memory enabled, without changes in PTV coverage.

### 3.4 Planning time

**FIGURE 5:**
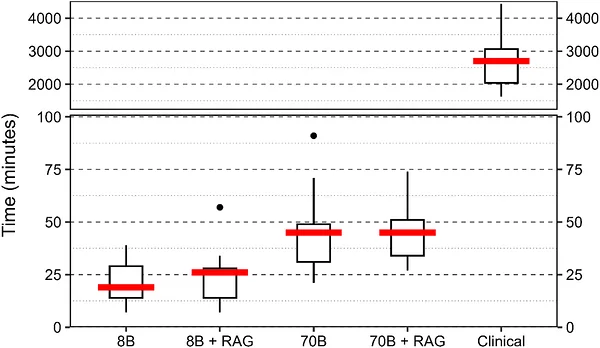
Boxplots display the distribution of total time for each SAGE variant and the clinical plans. Total time includes time required for all ten iterations per patient. Boxes indicate IQRs, medians are shown as horizontal lines, and whiskers extend to 1.5× IQR. Outliers are plotted as points. The y‐axis includes a break at y = 100 min and a scaling factor in order to visualize SAGE times compared to clinical planning times.

Planning times for SAGE were compared to dosimetrists’ planning time for clinical plans (Figure 5). SAGE planning time included the time required for it to conduct ten autonomous iterations of optimization in the TPS. The boxplot in Figure 5 depicts median times (red bar) as well as IQR (boxes) as well as outliers. Clinical planning times were obtained by measuring the time duration between start and end of the planning task in ARIA. These times were then corrected to remove non‐working hours and holidays for each patient. SAGE median times were: 19 min (8B; IQR: 14–29), 26 min (8B‐RAG; IQR: 14–28), 45 min (70B; IQR: 31–49), and 45 min (70B‐RAG; IQR: 34–51). The corrected dosimetrist median planning time was 2700 min (IQR: 2040–3060). In order to visualize this data, a y‐axis break and scaling factor (for the upper portion) was required in Figure 5.

## 4 DISCUSSION

We developed and evaluated SAGE, a locally deployed LLM agent for autonomous VMAT optimization‐priority tuning. Comparisons were made between retrospective, physician‐approved plans and four SAGE variants. While previous studies have utilized cloud‐based or proprietary models for planning tasks, this study evaluates a local open‐weight model deployment in which patient data remain within the institutional computing environment.

Enabling intra‐case memory produced a statistically significant improvement for a single endpoint, penile bulb V22Gy. Because both the clinical (44.8%) and 70B‐RAG (39.9%) arms already satisfied the protocol goal (< 50%), the clinical relevance of this difference is uncertain, and no statistically significant memory benefit was observed for the higher‐priority rectum or bladder endpoints. As summarized in Table 1 and tested statistically in Figure 3, SAGE balanced target coverage and OAR sparing relative to PROFIT protocol thresholds. No statistically significant difference in PTV V58.5 Gy coverage was observed between SAGE and the clinical plans, and the 70B‐RAG variant significantly reduced penile bulb V22Gy. Conversely, the clinical plans were statistically superior for rectum V20Gy and bladder V40Gy, although all SAGE 70B‐RAG values for these endpoints remained within PROFIT thresholds.

Statistical comparisons between SAGE plans and the clinical plans revealed nuanced performance trade‐offs. For OAR endpoints of intermediate dose spill (rectum V20Gy and bladder V40Gy), the clinical plans outperformed SAGE‐8B. This performance gap highlights a current limitation in the agent's action space. Human dosimetrists frequently employ auxiliary optimization structures (e.g., rings, tuning sectors) to strictly control dose fall‐off. In this study, SAGE was restricted to manipulating objective weights on anatomical structures alone.

Future iterations of SAGE will require the agent to generate and utilize auxiliary optimization structures (e.g., rings and tuning structures) to match human proficiency in controlling dose fall‐off and integral dose, as has been demonstrated in scripted and knowledge‐based automated planning workflows. The performance gap in rectum and bladder sparing is likely multifactorial. Beyond the restricted action space discussed above, differences in implicit prioritization between the agent and human dosimetrists may also contribute. Experienced dosimetrists bring clinical intuition about which OARs carry the greatest consequence for a given treatment site and may allocate disproportionate planning effort to rectum and bladder sparing for prostate cases.

Conversely, the advanced SAGE variant (70B‐RAG) achieved a statistically significant reduction in dose to the penile bulb while maintaining PTV coverage. This suggests that automated agents may strictly adhere to optimization objectives in ways human planners do not. In clinical practice, once mandatory constraints are met, further OAR sparing is pursued according to the As Low As Reasonably Achievable (ALARA) principle, balanced against practical limits such as available planning time and diminishing dosimetric returns for lower‐priority structures. The SAGE agent, driven by iterative feedback within a fixed ten‐iteration budget, continued to reduce penile bulb dose without compromising PTV coverage.

The memory‐enabled SAGE variants retrieve up to the three most recent prior priority choices and resulting DVH outcomes from the same patient's active optimization session; this memory store was reset between patients and did not persist across sessions. This is functionally similar to a trajectory buffer or episodic memory in closed‐loop agent optimization, and we do not claim it as a fundamentally novel learning mechanism. Prior LLM‐based radiotherapy planning studies have incorporated memory, historical plan retrieval, optimization trajectory analysis, and prior‐iteration feedback.18,20,35 Rather, the purpose of this component in the present study was to permit a controlled comparison between memory‐disabled and memory‐enabled agents while holding model family, action space, beam geometry, study‐defined objective definitions, priority initialization, and iteration count fixed. As seen in the results, enabling intra‐case memory improved SAGE's performance for penile bulb sparing, likely by reducing redundant priority choices and allowing the agent to avoid previously unsuccessful optimization strategies within the same patient session.

For the model size comparison, no statistically significant differences were noted. We expected larger models to perform better at treatment plan optimization due to improved reasoning capabilities and parameter counts.^30^, ^31^, ^32^, ^33^, ^34^, ^35^ The lack of significance suggests that for prostate geometries, which feature convex targets and relatively predictable OAR locations, the reasoning capabilities of the 8B model may be sufficient when augmented with intra‐case memory.

Planning times were calculated but not assessed statistically. As shown in Figure 5, planning times were mainly driven by model size. However, it is critical to distinguish between active compute time and clinical planning turnaround time. The corrected clinical planning duration (median 2700 min; IQR: 2040–3060) represents workflow turnaround time, including planning preparation, optimization, review, and non‐working intervals after correction for non‐working hours and holidays. While the active hands‐on time for a human dosimetrist is undoubtedly shorter than the full turnaround interval, the SAGE agent completed ten autonomous optimization iterations in a median of 45 min for the 70B‐RAG configuration. This speed may support operational efficiency by enabling rapid generation of candidate plans for human review. The systematic time difference between 70B and 8B variants is attributable to LLM inference latency. The 70B model requires proportionally longer per‐token generation time even when distributed across eight A100 GPUs. Both variants execute the same ten optimization iterations with identical TPS optimization and dose calculation settings.

Limitations of this work include the restriction to a single anatomical site. Prostate cancer planning involves generally convex targets; applying SAGE to complex geometries such as head and neck cancer, with concave targets and numerous serial OARs, will serve as a more stringent test of the agent's reasoning capabilities. SAGE's behavior reflects the combined effect of the LLM, fixed action space, TPS feedback loop, and prompt‐specified clinical prioritization rules; the system was not designed to learn treatment‐planning preferences de novo. It is hypothesized that the superior reasoning of larger models (70B+) may become statistically distinct from smaller models (8B) when navigating these complex, high‐dimensional trade‐off spaces.

This study evaluated optimized DVH endpoints only; plan deliverability was not assessed. We did not report monitor units, aperture‐ or modulation‐complexity metrics, or patient‐specific quality‐assurance results. Because optimization‐priority tuning can yield plans that appear acceptable on DVH endpoints yet are highly modulated or less robustly deliverable, the absence of a deliverability evaluation is a limitation of the present dosimetric comparison. Future work will involve a larger patient cohort spanning multiple anatomic sites and a rigorous deliverability evaluation, including monitor‐unit and plan‐complexity analysis and patient‐specific QA. Additionally, LLM outputs are stochastic at the non‐zero temperature used. Each patient‐variant plan was generated in a single run from identical initialization; repeated runs under identical initialization were not performed, so the run‐to‐run reproducibility of the final priority trajectories and dosimetric endpoints was not quantified. A repeatability analysis is a planned direction for future work.

We acknowledge that local deployment, while a practical and important step toward data governance, does not constitute a formal privacy‐preserving methodology such as differential privacy, federated learning, secure multiparty computation, or cryptographic privacy protection. Rather, it provides a privacy‐conscious deployment architecture that mitigates specific data‐governance risks: patient data are not transmitted to external model servers, reducing risks related to third‐party API logging, cloud provider data access, and data transfer outside institutional infrastructure.

The primary contribution of this study is a controlled 2 × 2 ablation that evaluates four agent variants and estimates the main effects of model scale and intra‐case memory under identical beam geometry, study‐defined objective definitions, priority initialization, and iteration limits. We do not claim the intra‐case memory mechanism or the local deployment strategy as fundamentally novel in isolation; rather, their value here lies in enabling this controlled comparison. SAGE is also built on an open‐weight model family, improving model‐level transparency and institutional deployability compared with proprietary closed model APIs. Finally, the use of PROFIT trial dosimetric criteria, rather than ad‐hoc institutional objectives, provides an externally interpretable benchmark for future comparisons.

SAGE illustrates a potential workflow automation model for radiotherapy planning. Rather than replacing dosimetrists, the eventual clinical role of such systems may be to support dosimetrists as supervisors of multiple planning agents. This could allow clinics to evaluate candidate plans more efficiently while preserving human oversight for plan review, clinical prioritization, and final approval. This work demonstrates that smaller locally deployed models, when augmented with intra‐case memory, can perform competitively for constrained prostate VMAT optimization‐priority tuning.

## 5 CONCLUSION

In this work, we demonstrated, as a proof‐of‐concept, that a locally deployed LLM agent can autonomously tune optimization priority values and generate clinically acceptable prostate VMAT plans within a fixed, study‐defined objective space. Intra‐case memory enhanced agent performance, while model size alone did not significantly improve the prespecified dosimetric endpoints. This work supports further investigation of language and vision models for radiation oncology automation, particularly in broader cohorts, more complex disease sites, and workflows with expanded action spaces.

## FUNDING INFORMATION

The authors have no funding sources to disclose for this work.

## CONFLICT OF INTEREST STATMENT

The authors declare that they have no conflicts of interest related to this work.

## Supporting information



Supporting Information

Supporting Information

## Data Availability

Prompt templates have been provided in the supplementary materials. Other data and code are available upon reasonable request to the authors.
