# Supplementary material for: Local large language model‐guided optimization for prostate radiotherapy planning
Source: Med Phys. 2026 Sep 10;53(9):e70654. doi: 10.1002/mp.70654 (PMC13559202; doi:10.1002/mp.70654)
Supplement: Supplementary file 1 [file MP-53-0-s001.pdf]

Supplementary Table S1. Representative SAGE ten-iteration optimization trajectory

Representative 70B-RAG case selected from the complete ten-iteration trajectory files. Iteration 0 reflects the neutral initial priority vector before SAGE priority tuning and should not be interpreted as a clinically optimized plan. Values marked with * did not meet the listed clinical-goal threshold. Clinical goals and optimizer objectives are related but not one-to-one; priority values are shown for SAGE's fixed action space only and are not compared with clinical-plan priority values.

Field	Value
**Representative case ID**	Representative case (anonymized)
**SAGE variant**	70B-RAG
**Prescription/fractionation**	60 Gy in 20 fractions
**Plan selected for analysis**	Plan generated at completion of the ten-iteration SAGE session
**Number of clinical goals/endpoints tracked**	19
**Number of study-defined optimizer objectives**	22

Panel A. DVH and clinical-goal evolution by iteration

Iteration	Goals met / total	PTV V58.5Gy (%)	Rectum V20Gy (%)	Rectum V60Gy (%)	Bladder V40Gy (%)	Bladder V60Gy (%)	Penile bulb V22Gy (%)	Notes
0	18/19	0.00*	29.34	0.00	0.00	0.00	27.14	Neutral initial priority vector; PTV coverage not met.
1	17/19	99.68	83.23	5.14*	9.98	1.84	36.21	Not met: Rectum V60Gy.
2	16/19	99.76	85.52*	5.62*	10.95	1.91	35.05	Not met: Rectum V20Gy, Rectum V60Gy.
3	18/19	99.79	82.25	5.15*	10.52	1.90	36.14	Not met: Rectum V60Gy.
4	18/19	99.63	71.77	4.58*	10.57	1.89	34.86	Not met: Rectum V60Gy.
5	18/19	99.60	50.14	3.96*	10.02	2.02	34.40	Not met: Rectum V60Gy.
6	18/19	99.56	56.42	3.99*	10.20	1.96	35.47	Not met: Rectum V60Gy.
7	18/19	99.44	43.77	3.45*	9.38	1.94	35.71	Not met: Rectum V60Gy.
8	18/19	99.52	38.77	3.38*	9.59	2.02	34.72	Not met: Rectum V60Gy.
9	18/19	99.47	45.12	3.24*	9.48	1.88	34.90	Not met: Rectum V60Gy.
10	18/19	99.44	39.71	2.99*	9.95	2.09	34.29	Not met: Rectum V60Gy.

**Indicates that the selected clinical goal was not met at that iteration.*

Panel B. Selected SAGE priority trajectories by iteration

Panel B shows selected high-yield objectives from SAGE's fixed action space. Panel C lists the full final priority vector.

Objective ID / optimizer objective	0	1	2	3	4	5	6	7	8	9	10
14. PTV High Risk: V60Gy>98%	1	150	180	180	160	180	160	150	180	160	180
15. PTV High Risk: V60Gy>100%	1	150	180	180	160	180	160	150	180	160	180
18. Rectum: V60Gy<1%	1	10	15	25	30	50	40	50	60	60	70

Objective ID / optimizer objective	0	1	2	3	4	5	6	7	8	9	10
19. Rectum: V50Gy<22%	1	10	15	25	30	50	40	50	60	50	60
20. Rectum: V40Gy<38%	1	10	15	25	30	50	40	50	60	50	60
21. Rectum: V20Gy<50%	1	5	10	15	20	40	30	40	50	40	50
3. Bladder: V60Gy<3%	1	10	12	20	15	20	25	30	40	30	40
5. Bladder: V48Gy<25%	1	10	12	20	15	20	25	30	40	30	40
6. Bladder: V40Gy<50%	1	10	12	20	15	20	25	30	40	30	40
8. Penile Bulb: V22Gy<50%	1	15	18	18	18	30	20	25	35	40	45

Panel C. Final SAGE priority vector for the representative case

Objective ID	Structure / ROI	Objective type	Fixed objective parameter(s)	Initial SAGE priority	Final SAGE priority	Final-iteration note
1	Bowel Small	Upper DVH objective	V40Gy<0%	1	20	Final value after ten SAGE iterations.
2	Bowel Small	Upper DVH objective	V33Gy<60%	1	20	Final value after ten SAGE iterations.
3	Bladder	Upper DVH objective	V60Gy<3%	1	40	Final value after ten SAGE iterations.
4	Bladder	Upper DVH objective	V56.8Gy<10%	1	40	Final value after ten SAGE iterations.
5	Bladder	Upper DVH objective	V48Gy<25%	1	40	Final value after ten SAGE iterations.
6	Bladder	Upper DVH objective	V40Gy<50%	1	40	Final value after ten SAGE iterations.
7	Bladder	Mean dose objective	Dmean<20Gy	1	30	Final value after ten SAGE iterations.
8	Penile Bulb	Upper DVH objective	V22Gy<50%	1	45	Final value after ten SAGE iterations.
9	Penile Bulb	Mean dose objective	Dmean<25Gy	1	25	Final value after ten SAGE iterations.
10	Femoral Head Left	Upper DVH objective	V35Gy<5%	1	20	Final value after ten SAGE iterations.
11	Femoral Head Left	Upper DVH objective	V37Gy<0%	1	20	Final value after ten SAGE iterations.
12	Femoral Head Right	Upper DVH objective	V35Gy<5%	1	20	Final value after ten SAGE iterations.
13	Femoral Head Right	Upper DVH objective	V37Gy<0%	1	20	Final value after ten SAGE iterations.
14	PTV High Risk	Lower DVH objective	V60Gy>98%	1	180	Final value after ten SAGE iterations.
15	PTV High Risk	Lower DVH objective	V60Gy>100%	1	180	Final value after ten SAGE iterations.
16	PTV High Risk	Upper DVH objective	V61.5Gy<0%	1	5	Final value after ten SAGE iterations.
17	PTV High Risk	Upper DVH objective	V61Gy<5%	1	5	Final value after ten SAGE iterations.
18	Rectum	Upper DVH objective	V60Gy<1%	1	70	Final value after ten SAGE iterations.
19	Rectum	Upper DVH objective	V50Gy<22%	1	60	Final value after ten SAGE iterations.
20	Rectum	Upper DVH objective	V40Gy<38%	1	60	Final value after ten SAGE iterations.
21	Rectum	Upper DVH objective	V20Gy<50%	1	50	Final value after ten SAGE iterations.
22	Rectum	Mean dose objective	Dmean<20Gy	1	50	Final value after ten SAGE iterations.
